# A remarkable beak morphology in a bird skull from the Eocene of Messel (Germany) signifies unusual feeding specializations

**DOI:** 10.1098/rsos.250620

**Published:** 2025-06-25

**Authors:** Gerald Mayr, Krister Smith

**Affiliations:** ^1^ Ornithological Section, Senckenberg Research Institute Frankfurt, Frankfurt am Main, Germany; ^2^ Department of Messel Research and Mammalogy, Senckenberg Research Institute Frankfurt, Frankfurt am Main, Germany

**Keywords:** *Aenigmatorhynchus rarus*, gen. et sp. nov., Aves, beak morphology, fossil birds, evolution

## Abstract

We report the skull of a new avian species from the latest early or earliest middle Eocene fossil site Messel in Germany. *Aenigmatorhynchus rarus,* gen. et sp. nov. is characterized by a long, straight, and pointed beak, as well as a mandible with prominent processus coronoidei, a very long symphysis, closely adjacent cristae tomiales, and a narrow dorsal sulcus along the tip. This unusual character mosaic impedes a straightforward phylogenetic assignment. In its proportions, the mandible is superficially similar to that of extant stilts (*Himantopus*, Recurvirostridae) and oystercatchers (*Haematopus*, Haematopodidae), but some features preclude an assignment of *Ae. rarus* to these and other charadriiform taxa. The ventral ossification of the rostrum suggests comparisons with long-beaked taxa of the Aequornithes and Telluraves, but again several features conflict with a position of *Ae. rarus* within either of these clades. Even though an unambiguous phylogenetic placement is not possible, the new fossil expands the avifauna of the Messel site and exhibits a distinctive beak morphology, which is not found in extant birds and indicates a specialized foraging behaviour as yet unknown in birds.

## 1. Introduction

The Messel Pit in Germany has yielded an abundant Eocene avifauna, which to date includes more than 70 species [1–3]. The fossiliferous horizons of the Middle Messel Formation are currently dated to around the early–middle Eocene boundary [4–6]. Even though the sediments of the locality are of lacustrine origin, they mainly preserve the remains of arboreal and terrestrial birds as well as those of aerial insectivores [2]. Fossils of clearly aquatic or semi-aquatic birds are very rare in Messel [2,7].

Apart from the superabundant ‘rail-like’ *Messelornis cristata*, whose ecology is not well known but which may have foraged in a nearshore environment [8], the best-represented species with a presumed semi-aquatic ecology is the ‘Messel ibis’ *Rhynchaeites messelensis*, of which a fair number of complete skeletons have been found [9,10]. The few other birds, which may have foraged in the immediate environment of the Messel Lake, are represented by single specimens in the bird inventory from Messel [2]. These include the long-legged *Juncitarsus merkeli*, which is a stem group representative of the flamingo/grebe clade [11,12], a putative stem group representative of the Suliformes (*Masillastega rectirostris*) [13], a putative charadriiform bird (*Vanolimicola longihallucis*) [14], and another small—unambiguously identified but as yet unnamed—charadriiform species [15].

Here, we report an isolated skull from Messel, which was found in the 2022 excavation campaign and exhibits a long and pointed beak that, in some aspects, superficially resembles the rostrum of the extant charadriiform taxa *Haematopus* (Haematopodidae) and *Himantopus* (Recurvirostridae). The fossil represents a new species and is a rare example of a well-preserved skull of a larger-sized bird from Messel, adding a distinctive new taxon to the avifauna of Messel. Even though its similarity to the skull of the Recurvirostridae and Haematopodidae may suggest a semi-aquatic ecology, the new species exhibits an unusual character mosaic, which impedes a straightforward phylogenetic placement and inferences about its foraging strategies.

## 2. Material and methods

The fossil is deposited in Senckenberg Research Institute Frankfurt, Germany (SMF). Comparisons with extant skeletons are based on the collection in SMF, except for *Ibidorhyncha struthersii*, which was examined on the basis of images in the Smithsonian online database at https://collections.nmnh.si.edu/search/birds/. Anatomical terminology follows Baumel & Witmer [16].

**Figure 1.**
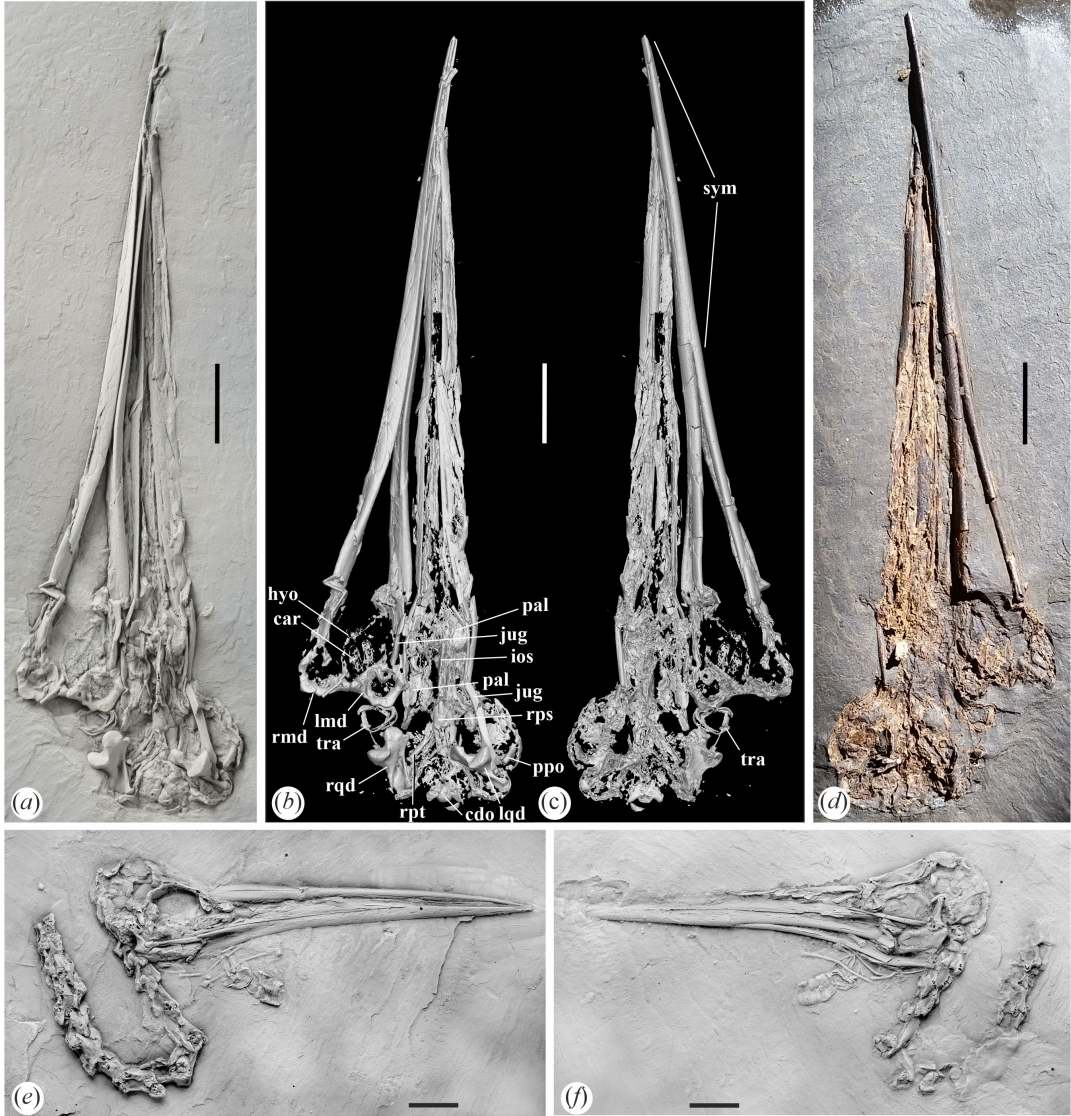
Skulls of *Aenigmatorhynchus rarus* from the latest early or earliest middle Eocene of Messel in Germany. (*a*) The holotype specimen (SMF-ME 11857A) coated with ammonium chloride. (*b*), (*c*) µCT scans of the holotype. (*d*) The holotype before it was transferred to artificial resin (photograph by Bruno Behr). (*e*), (*f*) a referred skull of *Ae. rarus* in the Pohl collection (PBP-MES−590A+B); coated with ammonium chloride. *Abbreviations*: car, cartilago arytenoidea; cdo, condylus occipitalis; hyo, hyoid apparatus; ios, ventral margin of interorbital septum; jug, jugal bar; lmd, articular (caudal) end of left mandibular ramus; lqd, left quadrate; pal, palatine (os palatinum); ppo, processus postorbitalis; rmd, articular (caudal) end of right mandibular ramus; rps, rostrum parasphenoidale; rpt, right pterygoid; rqd, right quadrate; sym, symphysis mandibulae; tra, tracheal rings. The scale bars equal 10 mm.

A µCT scan of the new skull, SMF-ME 11857 (figure 1*b,c*), was conducted in a customized version of the Tomo Scope XS Plus 200 (Werth Messtechnik, Gießen, Germany) at the Senckenberg Research Institute in Frankfurt. Parameters: 1 mm aluminium filter, 208 mA, 120 kV voltage, exposure time 500 ms, 2100 projections, 2× image averaging, 1×3 scan raster, no binning or drift correction, voxel resolution 17.637 μm. The scan of the skull of the holotype of *Juncitarsus merkeli*, SMF-ME 12000, was conducted on a TomoScope HV 500 (Werth Messtechnik, GmbH) in an industrial µCT facility funded by the Wolfgang Pfeiffer Stiftung at the Technische Hochschule in Deggendorf, Germany. Parameters: 130 mA, 190 kV, exposure time 666 ms, 1600 projections, no binning or drift correction, voxel resolution 25.332 μm. The volumes were imported to VG Studio Max (Volume Graphics, Germany) for volume rendering and study.

We note that there exists a second skull of this species in the collection of Dr Burkhard Pohl, which has the collection number PBP-MES-590A+B and also preserves part of the vertebral column (figure 1*e,f*). Size and morphology—especially the long mandibular symphysis—unambiguously support an assignment of this fossil to the new species. Because the specimen is not deposited in a public collection, it is not considered in the following, except for a comment on the shape of the nostrils, which are more clearly seen here than in the holotype.

## 3. Systematic palaeontology

### 3.1. Aves Linnaeus, 1758

### 3.2. Order and family incertae sedis

### 3.3. *Aenigmatorhynchus*, gen. nov.

#### 3.3.1. Type species

*Aenigmatorhynchus rarus*, sp. nov.

#### 3.3.2. Diagnosis

The new taxon is characterized by a long, straight, mediolaterally narrow and pointed beak, which reaches about 75% of the entire skull length; the mandibular symphysis is very long and measures about half the length of the mandible; the rami mandibularum run in parallel in the rostral half of the mandible; the mandible exhibits prominent processus coronoidei and its tip forms a trough-like dorsal sulcus. The latter two features probably represent autapomorphies of the new taxon.

#### 3.3.3. Etymology

The genus name is derived from *aenigma* (Lat.), riddle—which in turn is derived from *αἴνιγμα* (Gr.), speaking in riddles—and *ῥύγχος* (Gr.), beak; the taxon name refers to the unusual character distribution shown by the mandible of the new species.

### 3.4. *Aenigmatorhynchus rarus*, sp. nov.

#### 3.4.1. Holotype

**Figure 2.**
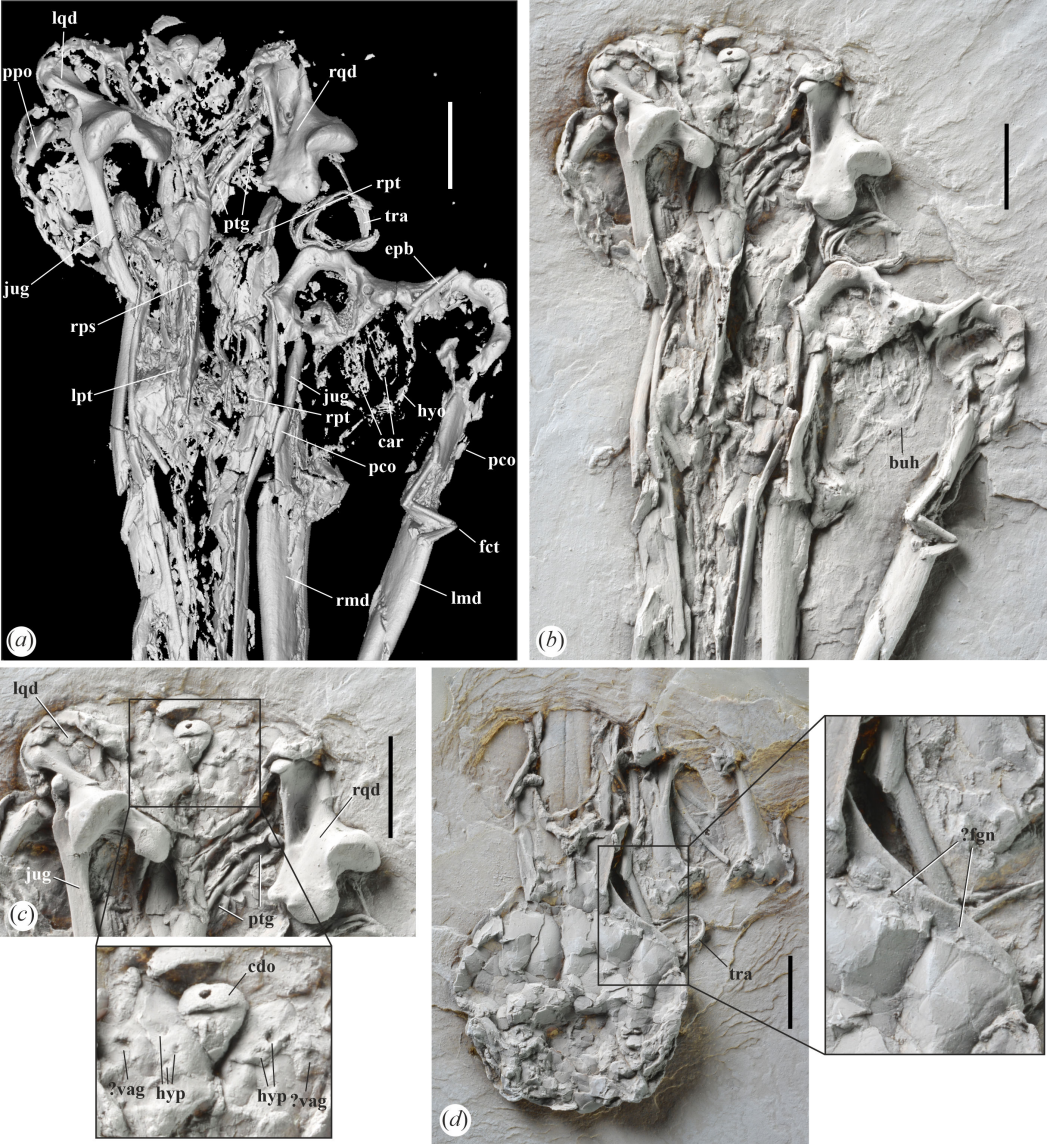
Skull details of *Aenigmatorhynchus rarus* (holotype, SMF-ME 11857A+B) from the latest early or earliest middle Eocene of Messel in Germany. (*a*), (*b*) Neurocranium (SMF-ME 11857A) in ventral view; (*a*) is a µCT scan, in (*b*) the specimen was coated with ammonium chloride. (*c*) Detail of caudal portion of neurocranium in ventral view; the specimen was coated with ammonium chloride; the framed area indicates a portion of the basicranial area that is shown in enlarged detail. (*d*) Neurocranium (SMF-ME 11857B) in dorsal view; the framed area indicates a portion of the orbital rim with a presumptive fossa glandulae nasalis that is shown in enlarged detail; the specimen was coated with ammonium chloride. *Abbreviations*: buh, os basiurohyale; car, cartilago arytenoidea; cdo condylus occipitalis; epb, os epibranchiale; fct, fracture; fgn, fossa glandulae nasalis; hyp, foramina nervi hypoglossi; jug, left jugal bar; lmd, left ramus mandibulae, lpt, left os palatinum; lqd, left quadrate; hyo, hyoid apparatus; pco, processus coronoideus; ppo, processus postorbitalis; ptg, right pterygoid; rmd, right ramus mandibulae; rps, rostrum parasphenoidale; rpt, right os palatinum; rqd, right quadrate; tra, tracheal rings; vag, foramen nervi vagi. The scale bars equal 5 mm.

SMF-ME 11857A and 11 857B (figures 1 and 2; isolated skull, main part and counterpart, respectively).

#### 3.4.2. Diagnosis

As for genus.

#### 3.4.3. Etymology

The species epithet is derived from rarus (Lat.), rare, in reference to the fact that the holotype skull currently is the only record of the species in a public collection.

#### 3.4.4. Type locality and horizon

Messel near Darmstadt, Germany; Middle Messel Formation, between 197 and 230 cm above marker bed Alpha, latest early or ealiest middle Eocene (circa 48 Ma [6]).

#### 3.4.5. Measurements

Skull length as preserved, 86.5 mm; estimated total length, approximately 90.0 mm. Rostrum, length, approximately 63 mm; estimated total length, approximately 66.5 mm. Mandible, length, 86.7 mm.

#### 3.4.6. Remarks

**Figure 3.**
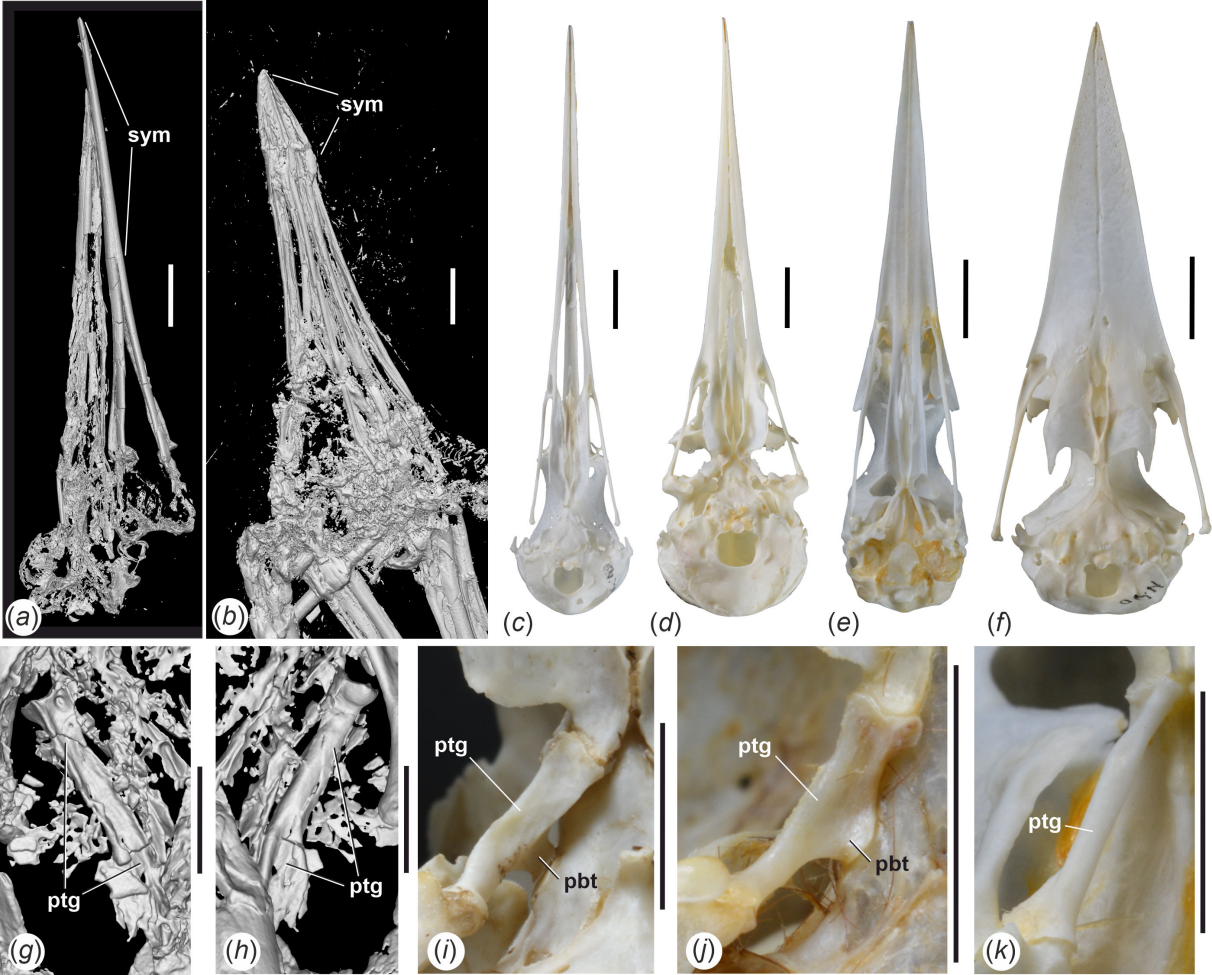
µCT scans of the skulls of (*a*) *Aenigmatorhynchus rarus* (holotype, SMF-ME 11857A) from the latest early or earliest middle Eocene of Messel in Germany and (*b*) *Juncitarsus merkeli* (holotype, SMF-ME 12000), also from Messel, which show the disparate lengths of the symphysis mandibulae. (*c*)‒(*f*) Skulls of extant long-beaked birds (ventral view) that illustrate different widths of the neurocranium: (*c*) *Himantopus mexicanus* (Recurvirostridae, SMF 16058), (*d*) *Haematopus ostralegus* (Haematopodidae, SMF 20208), (*e*) *Ixobrychus minutus* (Ardeidae, SMF 19846), (*f*) *Halcyon malimbica* (Alcedinidae, SMF 12795). (*g*)‒(*k*) Right pterygoid of (*g*), (*h*) *Ae. rarus* (holotype) in (*g*) dorsal and (*h*) ventral view, (*i*) *H. ostralegus* (SMF 5396, ventral view), (*j*) *H. mexicanus* (SMF 8929, ventral view), (*k*) *I. minutus* (SMF 19846, ventral view). *Abbreviations*: pbt, processus basipterygoideus; ptg, pterygoid; sym, symphysis mandibulae. The scale bars equal 10 mm for (*a*)‒(*f*) and 5 mm for (*g*)‒(*k*).

In its size and shape, the beak of the new species shows a resemblance to that of *Juncitarsus merkeli* (figure 3*b*), which is the only similar-sized bird from Messel with a long and straight beak. However, the mandible of *J. merkeli* has a proportionally much shorter symphysis and the rostrum is proportionally shorter, measuring only 62% of the total skull length. Accordingly, we dismiss the possibility that the new fossil belongs to *J. merkeli*.

#### 3.4.7. Description and comparisons

The skull has a long, straight, mediolaterally narrow and sharply pointed rostrum that measures about 80% of total skull length. The cristae tomiales form sharp ridges. Owing to the flattening of the specimen, the exact shape and extent of the nostrils cannot be determined in the holotype, but they do not reach into the rostral half of the rostrum (the referred specimen shows short and holorhinal nostrils). The maxillary bones appear to be co-ossified along most of their length. The ossa palatina are poorly preserved, but they are long and rectangular, and the right palatine bone seems to preserve a caudally projected angulus caudolateralis (figure 2*a*). The neurocranium is relatively small and mediolaterally narrow, of similar absolute and relative size to that of *Himantopus mexicanus* (Recurvirostridae). On its right side, there is a faint and narrow depression in the caudal portion of the frontal margin of the orbit (visible in SMF-ME 11857B), which may be a fossa glandulae nasalis (figure 2*d*). The interorbital section of the frontal bones is moderately wide. The ventral margin of the interorbital septum forms a sharp ridge. The processus postorbitalis is well developed and of subtriangular shape (figure 2*a*). Fossae temporales are not discernible owing to deformation of the corresponding area of the skull and appear to have been small and shallow. Likewise, there is no well-developed processus zygomaticus. The rostrum parasphenoidale is long; processus basipterygoidei are absent. Three (left side), respectively two (right side), foramina for nervus hypoglossus are well visible (figure 2*c*); identification of the foramina for n. vagus is tentative, and these may, alternatively, represent further passages for the hypoglossus nerve. The right pterygoid is preserved and is an elongate, rod-shaped element, which widens somewhat at the rostral end (figures 2*a* and 3*g,h*). The bone is proportionally more elongate than the pterygoid of the Haematopodidae (figure 3*i*) and Recurvirostridae (figure 3*j*). The condylus occipitalis is relatively small and caudally rather than ventrally facing, which may indicate a ‘Streckschädel’ [17], that is, an extended skull. The caudal portion of the jugal bar is mediolaterally flattened and bears a well-developed condylus quadraticus.

**Figure 4.**
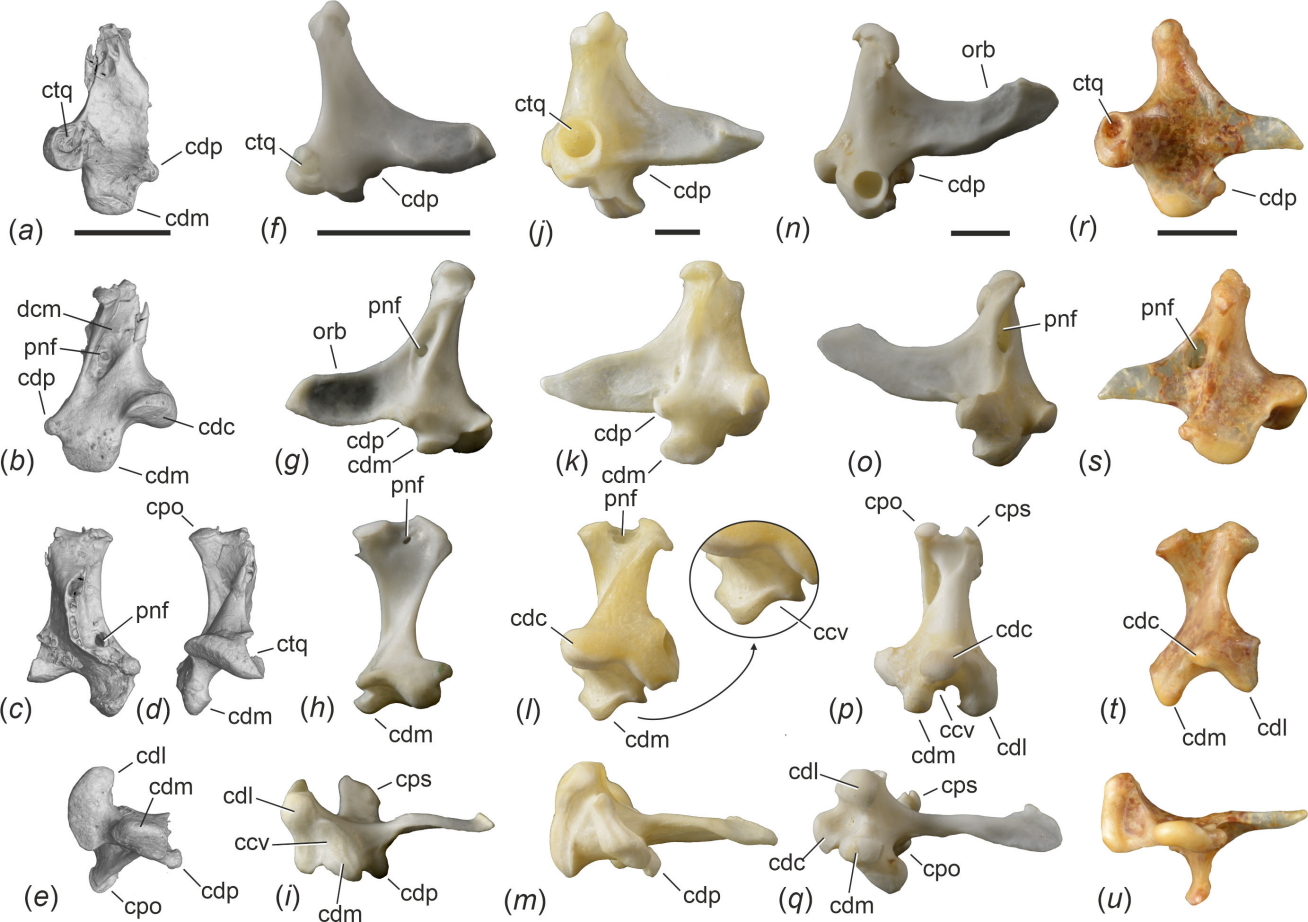
The fragmentary right quadrate of *Aenigmatorhynchus rarus* in comparison to the quadrates of selected extant species (first row: lateral view; second row: medial view; third row: caudal view [except for (*c*), which shows the bone in rostral view]; fourth row: ventral view). (*a*)‒(*e*) µCT scan of *Ae. rarus* (holotype, SMF-ME 11857A). (*f*)‒(*i*) *Himantopus mexicanus* (Recurvirostridae, SMF 16 048). (*j*)‒(*m*) *Mycteria leucocephala* (Ciconiidae, SMF 2847); the arrow indicates a detail of the condylus medialis. (*n*)‒(*q*) *Ardea cinerea* (Ardeidae, SMF 13 731). (*r*)‒(*u*) *Leptosomus discolor* (Leptosomidae, SMF 5438; left side, mirrored). *Abbreviations*: ccv, lateral concavity of condylus medialis; cdc, condylus caudalis; cdl, condylus lateralis; cdm, condylus medialis; cdp, condylus pterygoideus; cpo, capitulum oticum; cps, capitulum squamosum; ctq, cotyla quadratojugalis; dcm, depressio caudomedialis; orb, processus orbitalis; pnf, pneumatic foramen. The scale bars equal 5 mm.

**Figure 5.**
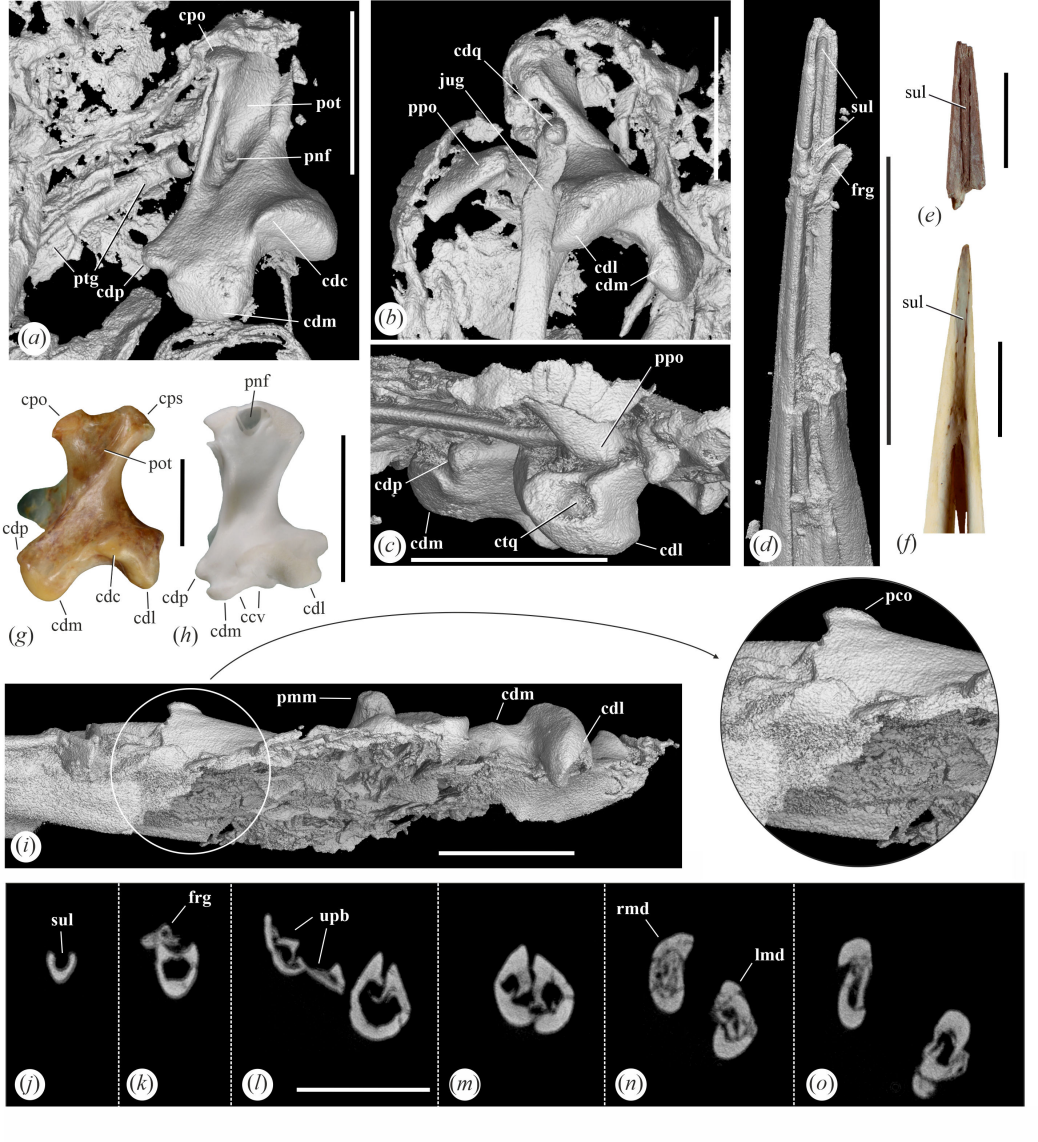
µCT scans of details of the quadrates and mandible of *Aenigmatorhynchus rarus* (holotype, SMF-ME 11857A) from the latest early or earliest middle Eocene of Messel in Germany. (*a*) *Ae. rarus*, right quadrate in caudomedial view. (*b*) *Ae. rarus*, left quadrate in caudoventromedial view. (*c*) *Ae. rarus*, ventral portion of left quadrate in lateral view. (*d*) *Ae. rarus*, tip of the mandible. (*e*) Tip of the mandible of *Matuku otagoense* (Ardeidae) from the early Miocene of New Zealand (see ref. [18]). (*f*) Tip of the mandible of the extant *Nycticorax caledonicus* (see ref. [18]). (*g*) Mirrored right quadrate of *Leptosomus discolor* (Leptosomidae, SMF 5438) in caudomedial view. (*h*) Left quadrate of *Himantopus mexicanus* (Recurvirostridae, SMF 16058) in caudomedial view. (*i*) *Ae. rarus*, caudal portion of left mandibular ramus in lateral view; the arrow denotes an enlarged detail showing the processus coronoideus. (*j*)–(*o*) *Ae. rarus*, cross sections through the mandible (from rostral to caudal). *Abbreviations*: ccv, lateral concavity of condylus medialis; cdc, condylus caudalis; cdl, condylus lateralis; cdm, condylus medialis; cdp, condylus pterygoideus; cdq, condylus quadraticus; cpo, capitulum oticum; cps, capitulum squamosum; ctq, cotyla quadratojugalis; frg, fragment of tip of mandible; jug, jugal bar; lmd, left ramus mandibulae; pco, processus coronoideus; pmm, processus medialis mandibulae; pnf, pneumatic foramen; pot, processus oticus; ppo, processus postorbitalis; ptg, pterygoid; rmd, right ramus mandibulae; sul, sulcus along dorsal surface of tip of mandible; upb, upper beak. The scale bars equal 5 mm.

Of both quadrates, the caudal portion is preserved (figures 4 and 5*a‒c*). Overall, the preserved parts of the bone show a resemblance to the quadrate of *Leptosomus* (Leptosomiformes; figures 4*r‒u* and 5*g*), but the condylus lateralis of the fossil is less ventrally prominent and the condylus pterygoideus better delimited from the condylus medialis. The processus oticus is mediolaterally broad and bears a depressio caudomedialis [19]; the µCT scan shows that at least the right quadrate exhibits a small pneumatic foramen in the ventral portion of this depression. Another pneumatic foramen is located on the rostromedial portion of the bone, dorsal to the condylus pterygoideus (figure 4*c*). Capitulum squamosum and capitulum oticum are well separated. The ventral surface of the condylus caudalis forms a shelf, which is continuous with the ventral surface of the condylus lateralis. The condylus lateralis forms a deep cotyla quadratojugalis (figure 5*c*). The condylus pterygoideus is well developed. Unlike in the Haematopodidae and Recurvirostridae (figure 4*f‒i*), the caudal surface of the tip of the processus oticus lacks pneumatic foramina. The condylus medialis is large and ventrally prominent; unlike in many extant Gruiformes, Charadriiformes (figure 5*h*), taxa of the Aequornithes (figure 4*l*) and others [20], its lateral surface does not form a concave articular facet.

**Figure 6.**
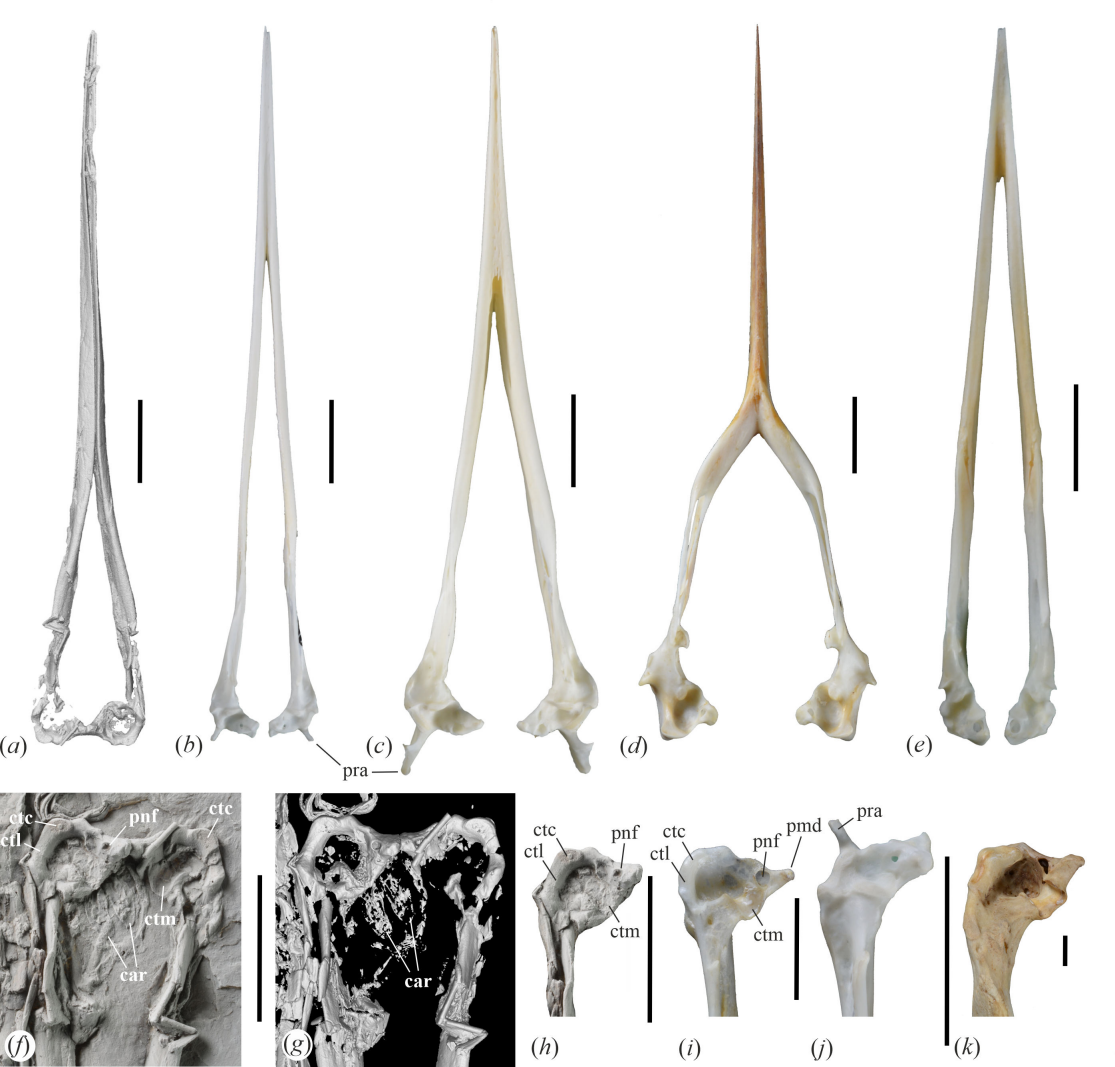
The mandible of *Aenigmatorhynchus rarus* (holotype, SMF-ME 11857A) from the latest early or earliest middle Eocene of Messel in Germany in comparison to that of extant long-beaked birds (dorsal view). (*a*) µCT scan of the mandible of *Ae. rarus*. (*b*) *Himantopus mexicanus* (Recurvirostridae, SMF 16058). (*c*) *Haematopus ostralegus* (Haematopodidae, SMF 20208). (*d*) *Rynchops niger* (Rynchopidae, SMF 4756). (*e*) *Ixobrychus minutus* (Ardeidae, SMF 19846). (*f*)–(*h*) detail of the caudal end of the mandible (dorsal view) of *Ae. rarus*; (*g*) is a µCT scan, in (*f*) and (*h*) the specimen was coated with ammonium chloride and in (*h*) the surrounding matrix was digitally removed. (*i*)–(*k*) detail of the caudal mandible (right ramus in dorsal view) of (*i*) *Leptosomus discolor* (Leptosomiformes, SMF 5438), (*j*) *H. mexicanus* (SMF 16058), and (*k*) *Balaeniceps rex* (Balaenicipitidae, SMF 1563). *Abbreviations*: car, cartilago arytenoidea; ctc, cotyla caudalis; ctl, cotyla lateralis; ctm, cotyla medialis; pmd, processus medialis; pnf, pneumatic foramen; pra, processus retroarticularis. The scale bars equal 10 mm.

The ventral surface of the skull is exposed on the main part (SMF-ME 11857A), whereas the detached mandible is seen in dorsal view. A zigzag-shaped fracture of the left mandibular ramus appears to be of perimortal origin; a straighter fracture of the right mandible transpired in almost the same position. The mandible of *Ae. rarus* is characterized by a very long and narrow symphysis, which measures about half the mandibular length. In the area of the symphysis, the co-ossified rami run in parallel, and the mandible is mediolaterally narrow. The cristae tomiales form sharp ridges; as preserved, they are inclined and meet each other in the midpart of the mandible, in the caudal section of the symphysis. This does not seem to be an artefact of preservation, because the µCT scans show the mandibular rami to be intact in cross sections (figure 5*j‒o*), and their sub-triangular dorsal portions are closely adjacent and separated by a narrow cleft. The rostralmost five millimetres of the dorsal surface of the mandible form a narrow, trough-like sulcus (figure 5*d*; the very tip of the mandible is broken and displaced). Caudally, this sulcus becomes shallower where it roofs the hollow lumen of the mandibular ramus (figure 5*k*). A symphyseal sulcus also occurs along the tip of a mandible from the Miocene of New Zealand (figure 5*e*), which was referred to the Ardeidae [18]; the authors of the latter study also figured a mandible of the extant *Nycticorax caledonicus* (Ardeidae) that shows a dorsal sulcus (figure 5*f*); we could not confirm its presence in other Ardeidae (including *Nycticorax nycticorax* and *Nyctanassa violacea*), but a dorsal sulcus appears to be present on the mandible of *Ibidorhyncha* (Ibidorhynchidae). The caudal portion of the mandibular ramus bears a prominent, rostrocaudally extensive processus coronoideus (figure 5*i*). Fenestrae mandibularum cannot be discerned. The caudal (articular) ends of the mandible are situated close together, which corresponds to the mediolaterally narrow neurocranium. The caudal end (figure 6*f‒h*) lacks a retroarticular process and resembles the caudal mandible of various only distantly related taxa, such as the Diomedeidae, Fregatidae, Ciconiidae and Balaenicipitidae (figure 6*k*) among the Aequornithes and the Tytonidae, Leptosomidae (figure 6*i*), and Trogonidae among the Telluraves. The caudal margin of the articular end is not dorsoventrally deep and truncate as it is in, for example, the Ciconiidae, but is dorsoventrally shallow and slanted; in dorsal view, its caudal margin is convex. Cotyla lateralis and cotyla medialis are merged into a common, shallow articular surface; the caudomedial portion of this facet is essentially flat and delimited by a small step from the remainder of the articular end. The cotyla medialis is wide and likewise shallow. The processus medialis is fairly short and somewhat dorsally deflected; the dorsal surface near its tip bears a foramen pneumaticum articulare.

For the first time, elements of the larynx could be identified in a bird from Messel, with the paired cartilagines arytenoideae being preserved in SMF-ME 11857A (figure 6*f,g*). The hyoid bones are also present and the basiurohyal (figure 2*a*) appears to have been short and wide. Ossified tracheal rings are situated next to the caudal end of the right mandibular ramus.

## 4. Discussion

*Aenigmatorhynchus rarus*, gen. et sp. nov. is characterized by its long, straight and pointed beak, the presence of well-developed processus coronoidei, and the unusually long mandibular symphysis, which bears a distinct dorsal sulcus along its tip. However, an assessment of the phylogenetic affinities of isolated Palaeogene bird skulls is sometimes less straightforward than that of some postcranial elements, and we could not identify derived characters, which would allow an unambiguous referral of *Ae. rarus* to an avian higher-level clade.

Very long, straight and pointed beaks evolved multiple times independently in neornithine birds and occur in species of the Podicipediformes, Charadriiformes (Recurvirostridae, Haematopodidae, Dromadidae, Rostratulidae, Scolopacidae, Sternidae, Rynchopidae), Gruiformes (Rallidae, Gruidae), Eurypygiformes (Eurypygidae), Strisores (Trochilidae), Aequornithes (Ardeidae, Ciconiidae, Anhingidae) and Telluraves (Upupiformes, as well as some Coraciiformes, Piciformes and Passeriformes). *Ae. rarus* can readily be differentiated from all of these extant taxa, but the new species from Messel may be a stem group representative of a taxon, which originated from a straight-beaked ancestor and subsequently evolved a different beak morphology (like the curved beak of the charadriiform Ibidorhynchidae and the gruiform Aramidae), or it may represent an extinct taxon without close extant relatives or with extant relatives that have short beaks.

As detailed above, *Ae. rarus* may have featured narrow fossae glandularum nasales, which suggests comparisons with the Charadriiformes. In its overall proportions and with regard to the long symphysis, the mandible of *Ae. rarus* resembles that of the extant charadriiform taxa *Himantopus* (Recurvirostridae) and *Haematopus* (Haematopodidae) (figure 6). However, unlike in these charadriiform birds and the closely related Ibidorhynchidae, the mandible of *Ae. rarus* lacks retroarticular processes (figure 6*h,j*) and exhibits pronounced processi coronoidei. There are further differences in the shape of the caudal end of the mandible (which has a larger and more roundish fossa articularis quadratica and a more convexly curved rostromedial margin in *Ae. rarus*), the pterygoid (which is much shorter in the Haematopodidae, Ibidorhynchidae, and Recurvirostridae and exhibits articulation facets for basipterygoid processes in these extant taxa), and the quadrate (which in *Ae. rarus*, among others, lacks pneumatic foramina on the caudal surface of the tip of the processus oticus and a markedly concave articular surface on the lateral surface of the condylus medialis). Furthermore, and unlike in the Recurvirostridae, Ibidorhynchidae and Haematopodidae, the rostrum of *Ae. rarus* has a thoroughly ossified ventral surface, and the mandibular rami run in parallel for more than half the total length of the mandible.

The morphology of the mandible of *Ae. rarus* also suggests comparisons with the charadriiform Rynchopidae, in which the mandibular rami are co-ossified in the rostral half and—unlike in the fossil species—form a dorsoventrally deep, blade-like structure (figure 6*d*). The mandible of the Rynchopidae lacks retroarticular processes, and the pterygoid is long and rod-like as it is in *Ae. rarus*. The close association of the mandibular rami in the fossil taxon may be interpreted as an early evolutionary stage of the highly derived condition of the rynchopid mandible. However, in the quadrate of the Rynchopidae—as well as in that of other Charadriiformes—condylus caudalis and condylus lateralis are well separated and do not form a continuous shelf. The closest extant relatives of the Rhynchopidae are the Sternidae and Laridae, in which the mandible exhibits a small caudal projection, which is absent in *Ae. rarus*. Even though the cotyla lateralis of the Rynchopidae forms a pronounced projection, a coronoid process is absent.

The lack of retroarticular processes and a basipterygoid articulation, as well as the long, rod-like pterygoid and the prominent processus coronoidei distinguish *Ae. rarus* from other long-beaked Charadriiformes (even in taxa, in which retroarticular processes are absent, such as most Lari, the caudal end of the mandible forms a small and often hook-shaped projection). The prominent processus coronoidei as well as the thoroughly ossified ventral surface of the rostrum differentiate the new species from the Gruiformes and Eurypygiformes, in which the maxillary bones are widely separated, thereby contributing to a movable, rhynchokinetic beak.

Ossification of the rostrum characterizes long-beaked species of the Telluraves (the clade including most small arboreal birds) and Aequornithes (the clade including the paraphyletic traditional ‘Ciconiiformes’ and ‘Pelecaniformes’). The quadrate of *Ae. rarus* shows a resemblance to that of extant Leptosomiformes (figure 5*g*), with which the fossil species also agrees in the shape of the caudal end of the mandible (figure 6*i*). However, in its skull proportions, *Ae. rarus* differs from all extant species of Telluraves, in which the neurocranium is proportionally larger and wider (figure 3*f*), presumably owing to a proportionally larger brain (see [21]). In most non-leptosomiform Telluraves – and all long-beaked species of this clade (Meropidae, Alcedinidae, Upupiformes, Galbulidae and several Passeriformes)—the caudal margin of the articular end of the mandible forms a more strongly convex bulge than in *Ae. rarus*.

Compared to equally long-beaked taxa of the Aequornithes, the rostrum of *Ae. rarus* most closely resembles that of the Ardeidae and Ciconiidae in its shape and relative length. However, apart from the long and solidly ossified rostrum, there are no unambiguous derived features that would support an assignment of *Ae. rarus* to either of these two taxa or to any other Aequornithes. Because the Ardeidae and Ciconiidae are well separated in current phylogenies [22–24], a long, straight beak either evolved independently in these two taxa or it is plesiomorphic for the Pelecanimorphae *sensu* ref. [25]. This latter clade includes the Ciconiidae, Threskiornithidae, Ardeidae, Scopidae, Balaenicipitidae, Pelecanidae and Suliformes. In all of these taxa, the rostrum is very long and has a ventrally ossified surface. However, the Scopidae, Balaenicipitidae and Pelecanidae, which form a clade within Aequornithes, are characterized by a very short pterygoid unlike that of *Ae. rarus*, and the rostrum of these three extant taxa bears a marked terminal hook. Even though we note that the rostralmost tip of the rostrum of the *Ae. rarus* holotype is broken, a terminal rostral hook appears to have been absent, which is confirmed by the referred specimen and distinguishes the fossil species from all Suliformes other than the Anhingidae (which differ from *Ae. rarus* in the morphology of the quadrate and the caudal end of the mandible).

The oldest unambiguously identified fossils of the Ardeidae stem from early Oligocene sites in Europe and Asia [26,27], and the earliest unambiguous record of the Ciconiidae is from the early Oligocene of Egypt [27]. Being nearly 15 million years older, *Ae. rarus* may, therefore, be an archaic stem group representative of one of these clades. However, the quadrate of *Ae. rarus* shows a fairly generalized morphology and does not exhibit the derived shape characterizing the Ardeidae [19]; the quadrate of the Ciconiidae likewise differs from that of the fossil species. In contrast to the Ardeidae (figure 4*n‒q*), Ciconiidae (figure 4*j‒m*) and most other species of the Aequornithes except for the Pelecanidae and some Suliformes, the lateral surface of the condylus medialis does not form a marked concave articular facet and the ventral surfaces of condylus caudalis and condylus lateralis form a continuous shelf, whereas they are better delimited in most Aequornithes. Quadrate morphology therefore conflicts with a position of *Ae. rarus* within the Aequornithes. The caudal end of the mandible of the new species is also clearly distinguished from that of extant Ardeidae (which, for example, has a much more concave cotyla lateralis; figure 6*e*); the Ciconiidae have a relatively unspecialized caudal mandible similar to that of *Ae. rarus*.

Even though we are unable to constrain the phylogenetic affinities of *Ae. rarus*, the species adds a distinctive new taxon to the Messel avifauna. Furthermore, the combination of a long beak with prominent processus coronoidei indicates a specialized foraging behaviour unknown in any other extinct or extant bird.

The species of the charadriiform taxon *Himantopus* forage by probing, plunging and pecking, whereas those of *Haematopus* use their beak to prise bivalves and to probe for worms and other evertebrates [28–30]. Probing for food involves gaping, that is, the opening of the beak against substrate (e.g. soil or mud). The lack of retroarticular processes indicates that *Ae. rarus* was not employing this foraging strategy. Instead, the strongly developed processus coronoidei, which probably served for the attachment of musculus adductor mandibulae externus, pars rostralis, suggest that the morphological specializations of the new species enabled forceful closing—rather than opening—of the beak. It may be worth noting in this context that whereas gastropods are not uncommon at Messel, bivalves are unknown [3].

A well-developed coronoid process is a feature, which among extant birds mainly occurs in seed-eating, ‘finch-beaked’ passerines and other granivores (e.g. *Thinocorus*, Thinocoridae), as well as in filter-feeding Anseriformes and flamingos (Phoenicopteriformes) and a few other taxa with comparatively short beaks (*Cariama cristata* [Cariamidae], *Opisthocomus hoazin* [Opisthocomidae]). By contrast, in extant birds with beaks that are as long as that of *Ae. rarus* the mandibles lack prominent processi coronoidei, although the latter are developed as distinct ridges in some taxa, such as the Ardeidae and Ciconiidae. The occurrence of prominent coronoid processes in a very long-beaked bird like *Ae. rarus* is unexpected, because the forces exerted by the adductor muscle mainly acted on the caudal portion of the mandible and decreased towards the tip of the long beak. This suggests that the new species fed on hard food items, which may have been picked up with the long beak and cracked in the caudal section of the cristae tomiales.

## Data Availability

The image stack of SMF-ME 11857A and a surface mesh of its segmented right quadrate are made available at Zenodo [31]. All other relevant data are within the manuscript.
